# Multilevel Interactions of Stress and Circadian System: Implications for Traumatic Stress

**DOI:** 10.3389/fpsyt.2019.01003

**Published:** 2020-01-28

**Authors:** Agorastos Agorastos, Nicolas C. Nicolaides, Vasilios P. Bozikas, George P. Chrousos, Panagiota Pervanidou

**Affiliations:** ^1^Department of Psychiatry, Division of Neurosciences, Faculty of Medical Sciences, School of Medicine, Aristotle University of Thessaloniki, Thessaloniki, Greece; ^2^VA Center of Excellence for Stress and Mental Health (CESAMH), VA San Diego Healthcare System, San Diego, CA, United States; ^3^First Department of Pediatrics, Division of Endocrinology, Metabolism and Diabetes, School of Medicine, National and Kapodistrian University of Athens, “Aghia Sophia” Children’s Hospital, Athens, Greece; ^4^Unit of Developmental & Behavioral Pediatrics, First Department of Pediatrics, School of Medicine, National and Kapodistrian University of Athens, “Aghia Sophia” Children’s Hospital, Athens, Greece

**Keywords:** circadian system, circadian clocks, stress, trauma, HPA axis, autonomic nervous system, glucocorticoids, sleep

## Abstract

The dramatic fluctuations in energy demands by the rhythmic succession of night and day on our planet has prompted a geophysical evolutionary need for biological temporal organization across phylogeny. The intrinsic circadian timing system (CS) represents a highly conserved and sophisticated internal “clock,” adjusted to the 24-h rotation period of the earth, enabling a nyctohemeral coordination of numerous physiologic processes, from gene expression to behavior. The human CS is tightly and bidirectionally interconnected to the stress system (SS). Both systems are fundamental for survival and regulate each other’s activity in order to prepare the organism for the anticipated cyclic challenges. Thereby, the understanding of the temporal relationship between stressors and stress responses is critical for the comprehension of the molecular basis of physiology and pathogenesis of disease. A critical loss of the harmonious timed order at different organizational levels may affect the fundamental properties of neuroendocrine, immune, and autonomic systems, leading to a breakdown of biobehavioral adaptative mechanisms with increased stress sensitivity and vulnerability. In this review, following an overview of the functional components of the SS and CS, we present their multilevel interactions and discuss how traumatic stress can alter the interplay between the two systems. Circadian dysregulation after traumatic stress exposure may represent a core feature of trauma-related disorders mediating enduring neurobiological correlates of trauma through maladaptive stress regulation. Understanding the mechanisms susceptible to circadian dysregulation and their role in stress-related disorders could provide new insights into disease mechanisms, advancing psychochronobiological treatment possibilities and preventive strategies in stress-exposed populations.

## Introduction

Living organisms consist of highly complex biological systems with the ability to preserve a complex dynamic balance state with a constant oscillation around an ideal homeostatic condition (*nonequilibrium homeodynamic state*) (1, 2). To achieve this, organisms have developed a highly sophisticated and multifaceted biological system, the so-called stress system (SS), which serves self-regulation and adaptability of the organism to ongoing intrinsic or extrinsic, real or perceived (i.e., subject-dependent value attribution), altering challenges or stimuli, defined as stressors (3). When stressors surpass a manageable severity or temporal verge, the initiated stress response redirects energy depending on the present needs to restore homeostasis (4–8). Thus, stress is defined as the state of threatened homeodynamic balance of the organism (6, 9). Repeated, ephemeral, and motivating stress states lead to adaptive responses and are fairly beneficial, while inadequate, aversive, excessive, or prolonged stress may surpass the natural regulatory capacity and adjustive resources of the organism and majorly affect adaptive responses leading to *cacostasis (i.e., negatively altered homedynamic state, dyshomeostasis)*, and accumulated *cacostatic* load (6).

The understanding of the temporal relationship between stressors and physiological stress responses is crucial for the comprehension of the molecular basis of physiology and pathophysiology of disease. Biological processes always take place in an appropriate order, in order to synchronize required homeostatic mechanisms. As life on earth has evolved in the context of the earth’s rotation around its own axis, there was a geophysical evolutionary need for temporal organization and adjustment of internal activity and physiological processes to the dramatic fluctuations in energy demands by the constant rhythmic succession of night and day. This need has generated a highly conserved and sophisticated internal molecular “clock,” creating endogenous rhythmicity with a period adjusted to the 24-h rotation of our planet throughout phylogeny (10–12).

This intrinsic circadian (lat. *circa diem* – about a day) timing system (CS) creates an internal representation of the external *Zeitraum* (germ. time-space) and helps living organisms keep track of time from a centrally created circadian rhythm (13, 14). By orchestrating a dynamic milieu that oscillates with a 24-h rhythm, the CS coordinates physiological processes and rhythmic changes, from gene expression to behavior and prepares living organisms for the anticipated cyclic challenges, promoting homeostasis and environmental adaptation and creating an evolutionary advantage to optimize survival (15–18). In order to achieve this, the CS upregulates the SS before the organism’s active phase and turns it down again for the resting and restorative phases.

The CS and the SS are both fundamental for survival and regulate each other’s activity, through intimate reciprocal interactions with each other at multiple levels (19, 20). An intact communication between the CS and the SS is important for maintaining homeostasis and environmental adaptation (21–23). The SS is undoubtedly at the heart of circadian biology, mediating temporal signals and *vice versa* (24). Investigating the interactions between the two systems is essential to understand pathophysiological pathways mediating risk for disease, as dysregulation in either of these systems may lead to similar pathologic conditions (25).

In this review, following a general overview of the functional elements of the two systems, we present their multilevel interconnections, and discuss how excessive (i.e., traumatic) stress can affect the harmonic central and peripheral interplay between SS and CS.

## The Human Stress System

**Figure 1:**
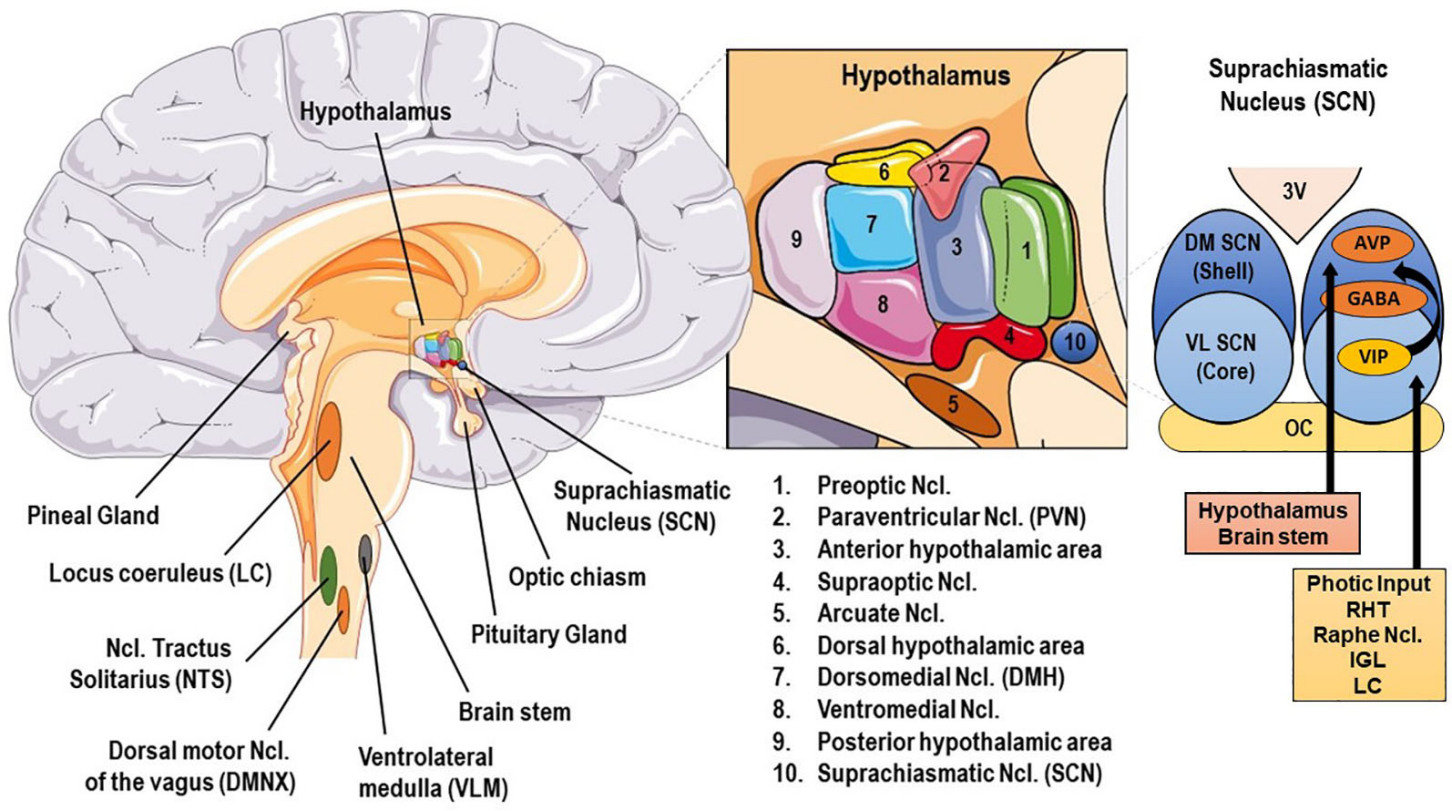
Basic anatomy of stress and circadian system related brain structures. AVP, arginine vasopressin; GABA, γ-aminobutyric acid; DM SCN, dorsomedial suprachiasmatic nucleus; IGL, thalamic intergeniculate leaflet; LC, locus caeruleus; RHT, retinohypothalamic tract; VIP, vasoactive intestinal peptide; VL SCN, ventrolateral SCN.

The human SS consists of central and peripheral components. The central, critically interconnected components of the SS are mainly located in the hypothalamus and the brainstem, and include: (a) the parvocellular neurons of corticotropine-releasing-hormone (CRH), (b) the arginine-vasopressin (AVP) neurons of the hypothalamic paraventricular nuclei (PVN), (c) the CRH neurons of the paragigantocellular and parabranchial nuclei of the medulla and the locus caeruleus (LC), (d) the arcuate nucleus proopiomelanocortin-derived peptides alpha-melanocyte–stimulating hormone (MSH) and beta-endorphin, (e) other mostly noradrenergic (NE) cell groups in the medulla and pons (LC/NE system), and (f) the central nuclei of the autonomic nervous system (ANS) [cf. **Figure 1**]. These neuroanatomical loci communicate with each other, influencing their own activity, and interact with several other brain subsystems, such as the mesocortical/mesolimbic dopaminergic system, involved in reward and motivation and the amygdala central nuclei, generating fear and anger (6, 9).

The peripheral components of the SS include: (a) the hypothalamic-pituitary-adrenal (HPA) axis and (b) the ANS comprised of (i) the sympathetic nervous system (SNS) and sympatho-adrenomedullary (SAM) system and (ii) the parasympathetic nervous system (PNS). The main terminal peripheral effector molecules of the SS are the HPA axis-regulated glucocorticoids (GCs; i.e., cortisol in humans), and the SAM-regulated catecholamines (Cas; i.e., NE and epinephrine). HPA axis and ANS have largely complementary actions throughout the body and are increasingly studied together (26), as integrated and interrelated components of an internal neural regulation system. Findings suggest that the appropriate regulation of the HPA-axis depends in part on ANS, especially on vagal influences (27).

When stressors exceed a certain severity or temporal threshold, stressor-related information initiates a complex stress response to induce remarkably consistent acute, normally adaptive, and time-limited microphysiologic, mesophysiologic, and macrophysiologic compensatory responses throughout several effector tissues (4–8, 28). Together, these responses represent a well-orchestrated and fine-tuned answer to challenge in both the central nervous system (CNS) and the somatic periphery (29).

### The Autonomic Nervous System

The ANS, although not under overt voluntary direction (*autonomous*), plays a crucial role in the preservation of a homeodynamic balance by providing a rapidly responding control system for a plethora of physiological reactions to physical, emotional, and cognitive challenges (30, 31). It is especially the precise regulation of organ and tissue functions through fine-tuning of the ANS limbs that is crucial for optimal stress reactivity, adaptive responses, and health.

**Figure 2:**
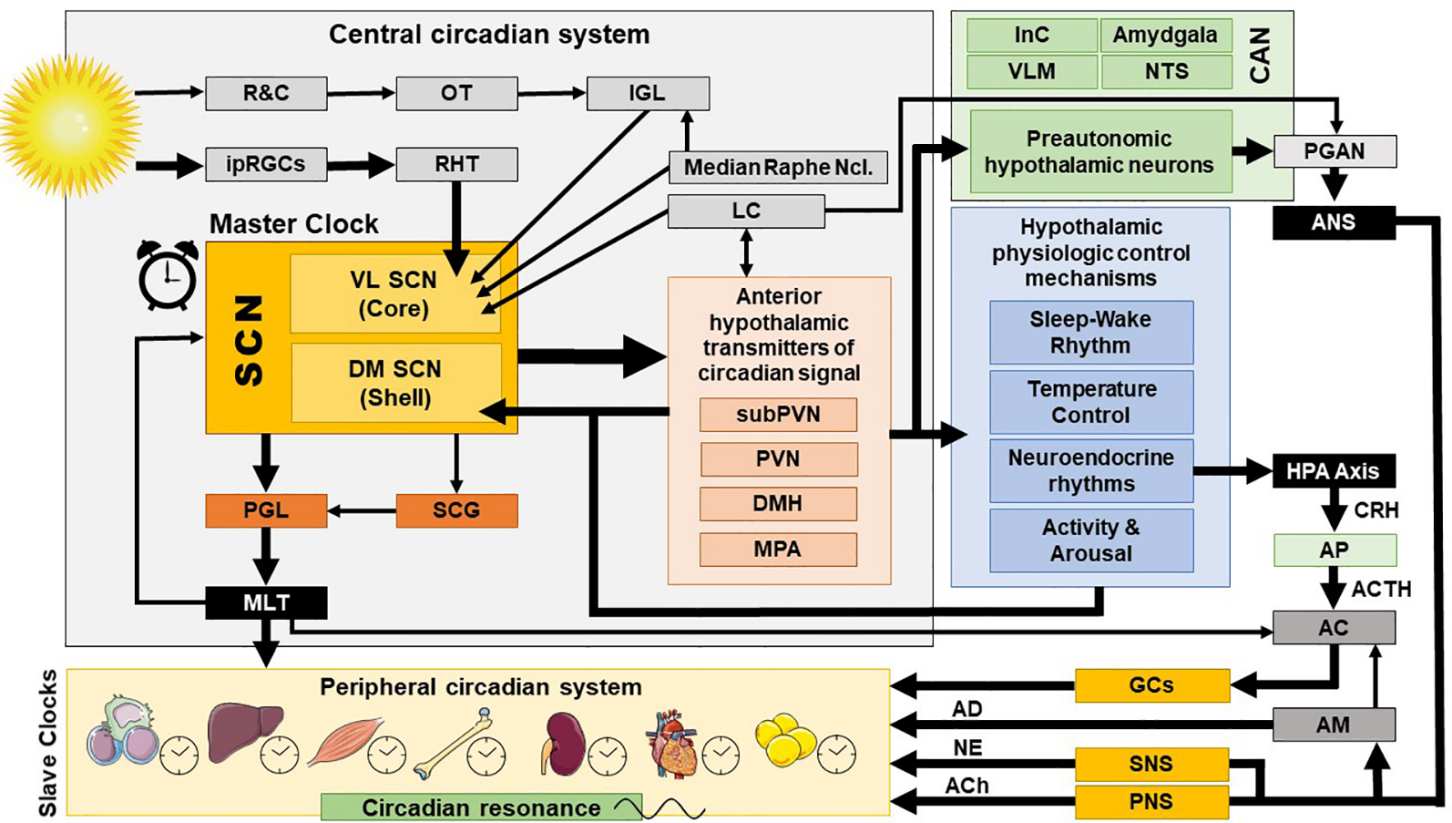
Central and peripheral circadian system and their interconnections. AC, adrenal cortex; ACh, acetylcholine; ACTH, adrenocorticotropic hormone; AD, adrenalin; AM, adrenal medulla; ANS, autonomic nervous system; AP, anterior pituitary; CAN, central autonomic network; CRH, corticotropin releasing hormone; DM SCN, dorsomedial SCN; DMH, dorsomedial hypothalamus; GCs, glucocorticoids; HPA axis, hypothalamic-pituitary-adrenal axis; InC, insular cortex; IGL, thalamic intergeniculate leaflet; ipRGC, intrinsically photosensitive retinal ganglion cells; LC, locus caeruleus; MLT, melatonin; MPA, medial preoptic area; NE, norepinephrine; NTS, nucleus of the solitary tract; OT, optic tract; PNS, parasympathetic nervous system; PGAN, preganglionic autonomic neurons; PGL, pineal gland; PVN, paraventricular nucleus; R&C, rodes and cones; RHT, retinohypothalamic tract; SCN, suprachiasmatic nucleus; SCG, superior cervical ganlia; SNS, sympathetic nervous system; subPVN, subparaventricular area; VL SCN, ventrolateral SCN; VLM, ventrolateral medulla.

The exact ANS activity is fine-tuned through central and peripheral autonomic reflexes and feedback mechanisms (32). The central autonomic modulation does not simply rely on a monolithic network of brain regions, but is instead regulated by the central autonomic network (CAN), an internal central autonomic regulation system featuring certain task and division specificity (33). The CAN is additionally characterized by bilateral interconnections, parallel organization, state-dependent activity, and neurochemical complexity (30, 31, 34, 35). It includes the insular cortex, central nucleus of the amygdala, hypothalamus, periaqueductal gray matter, parabrachial complex, nucleus of the solitary tract (NTS), and ventrolateral medulla (VLM) (36, 37) [cf. **Figure 2**]. The insular cortex and amygdala mediate high-order autonomic control associated with cognitive perception and emotional responses through hypothalamic-brainstem pathways (30). NTS, PVN, and VLM contain a network of respiratory, cardiovagal, and vasomotor neurons, receiving afferent vagal sensory input from thoracic and abdominal viscera and other cranial nerves. These structures accordingly modulate the activity of preganglionic autonomic neurons. CAN dysregulation can be critically involved in stress-related disorders, as it may affect downstream autonomic centers, thereby altering peripheral ANS activity and cardiac function. CAN dysregulation (35, 38, 39) may affect downstream autonomic core centers, thereby altering peripheral ANS activity (39–41).

Since the early 20th century, pragmatic and anatomic reasons has led to a common division of the ANS into two, or sometimes three peripheral tracts: the sympathetic, parasympathetic and, the largest one, the enteric autonomic division, although they practically mirror one larger control system (42, 43). Especially the separation into SNS and PNS has led to enormous misconceptions, the most serious being the view that the two divisions are somehow in opposition to each other. On the contrary, SNS and the PNS are rather in a dynamic *interdependent* state and act on different time scales but in concert and through numerous and multilevel, bidirectional interactions to control the abovementioned autonomic functions (44, 45), while autonomic dysregulation translates into decreased dynamic adaptability, increased morbidity and mortality (27, 30, 46, 47). In general, since both systems are tonically active, the PNS can both assist and antagonize SNS functions by withdrawing or increasing its activity (frequency of neuronal discharge), respectively. This ANS characteristic is of major importance and improves its ability to more precisely regulate an effector’s function.

#### The Sympathetic Nervous System

The SNS originates in brainstem nuclei and gives rise to preganglionic cholinergic (ACh) efferent fibers mostly projecting to postganglionic sympathetic ganglia. The long postganglionic neurons terminate outwards on effector tissues, mostly releasing NE. Alternatively, preganglionic neurons may also directly synapse with the modified postganglionic chromaffin cells of the adrenal medulla. A sympathetic activation, thus, principally releases NE (locally and to a lesser extent systematically from the adrenal medulla) or adrenaline (systematically from the adrenal medulla) together with other neuropeptides in the body (48). Sympathetic activation generally predominates during emergency (*fight-or-flight*) situations and during exercise, preparing the body for strenuous physical activity.

#### The Parasympathetic Nervous System

Whereas SNS activity depends on two peripheral branches (neural and adrenal), parasympathetic activity is displayed only by nerves. The preganglionic neurons of the PNS arise from numerous brainstem nuclei and from the spinal sacral region (S2–S4). The preganglionic ACh-axons are quite long and synapse with short postganglionic neurons within terminal ganglia close to or embedded to effector tissues. Accordingly, PNS actions are mostly more discrete and localized compared to the SNS, where a more diffuse and global discharge is probable. The preganglionic neurons that arise from the brainstem exit the CNS through the cranial nerves [N. occulomotorius (III); N. facialis (VII); N. glossopharyngeus (IX); N. vagus (X)]. The vagus nerve innervates the thoracic and abdominal viscera and has a major physiological significance, as approximately ¾ of all parasympathetic fibers originate from the vagus nerve (49). The PNS stress response is mainly activated by the nucleus ambiguus and the dorsal motor nucleus of the vagus nerve, possibly after NTS stimulation. The PNS generally predominates during resting conditions towards conserving and storing energy or regulating basic body functions (e.g., digestion, defecation, urination). Through its tonic properties, the PNS is vital especially under resting conditions, and is, therefore, particularly implicated in the development of cardiovascular diseases and other comorbidities (27, 50).

### The Hypothalamus-Pituitary-Adrenal Axis

The HPA axis consists of the PVN, the pituitary corticotrophs and the *zona fasciculata* of the adrenal cortex, which, respectively, employ corticotropin-releasing hormone (CRH)/arginine vasopressin (AVP), adrenocorticotropic hormone (ACTH), and glucocorticoids (GCs, i.e., cortisol in humans) as their signalling effector molecules [cf. **Figure 2**]. CRH and AVP are released from the PVN into the hypophyseal system in response to stimulatory signals from higher regulatory centers (e.g., PFC) and reach the pituitary gland to stimulate the secretion of ACTH. ACTH reaches the cortex of the adrenal glands through release in the systemic circulation and stimulates both production and secretion of GCs. Systemically released GCs, in turn, besides their major actions, close a negative feedback loop by suppressing the activation of the PVN and the pituitary gland (6, 51).

#### Glucocorticoid Receptors and Signaling

GCs influence a myriad of physiologic functions and are essential for the activation, maintenance, and downregulation of the stress response. GCs mainly exert their pleiotropic effects through genomic, nongenomic, and mitochondrial actions of the intracellular cognate GC and mineralocorticoid receptors (GR, MR), which function as a ligand-activated transcription factors (4–9, 52–56). GR and MR are evolutionarily close, showing large homologies at their DNA-binding domain and sharing many responsive genes. Upon ligand-binding, the receptors dissociate from the interacting proteins (i.e., shock proteins and immunophillins), translocate to the nucleus, form homo- or hetero-dimmers and bind to specific DNA response elements located in the regulatory regions of thousands responsive genes, leading to their transactivation or transrepression (8, 52, 54–57). GR and MR have complementary actions with respect to HPA axis activity and reactivity (58). Altered GC-signaling, through dysregulations at different levels of the HPA axis, may greatly negatively affect the organisms’ physiology and could influence life expectancy, as seen in many complex behavioral and somatic disorders (e.g., depression, posttraumatic stress disorder, sleep disorders, chronic pain and fatigue syndromes, obesity, diabetes Type II and the metabolic syndrome, essential hypertension, atherosclerosis, osteoporosis, autoimmune inflammatory, and allergic disorders) (55, 59).

In humans, the glucocorticoid receptor (hGR) is encoded by the *NR3C1* gene, which is located in the long arm of chromo-some 5 and consists of 10 exons. The alternative usage of exon 9α or 9β gives rise to the two main receptor isoforms, the classic hGRα and the hGRβ (8, 52, 54–57). Ubiquitarilly expressed in every tissue except the suprachiasmatic nucleus (SCN) of the hypothalamus, the hGRα is primarily localized in the cytoplasm of glucocorticoid target cells (57, 60). hGRβ, exclusively localized in the nucleus of certain cells (e.g., endothelial cells), acts mainly as negative regulator of hGRα transcriptional activity (61, 62). A growing body of evidence suggests that hGRβ has its own, hGRα-independent transcriptional activity and plays an important role in insulin signalling, inflammation, and carcinogenesis (63). The MR is encoded by the *NR3C2* gene, is located on chromosome 4 and also consisting of 10 exons (64). MR is peripherally expressed in several tissues (e.g., adipose tissue, kidney, endothelium, macrophages) and exerts vital regulatory functions through its main endogenous MR ligand as part of the renin-angiotensin-aldosterone system, among others, in cell growth, renal and cardiovascular function, metabolism and immunity.

Of particular importance are the GR and MR effects in the CNS. While GR are expressed throughout the brain, MR are abundantly expressed in limbic brain structures involved in emotional processing, arousal and memory (i.e., hippocampus, amygdala, prefrontal cortex) thus exerting a basal inhibitory tone on GC secretion (65, 66). Interestingly, the MRs show a tenfold higher affinity to cortisol than GRs and are largely already occupied under basal cortisol levels, while GRs become gradually occupied through cortisol peak levels (e.g., circadian peak, acute stress) (58, 67), resulting in a regulative, MR-associated threshold for HPA axis activation and stress sensitivity (68). Thus, depending on receptor type, cell topology, tissue-specific expression, their specific ligands (e.g., aldosterone) or relevant enzymes (e.g., cortisol-inactivating enzyme 11β-hydroxysteroid dehydrogenase type 2, 11βHSD2), HPA axis activation differentially regulates the expression of various target genes with different transcriptional potencies in response to cortisol.

In addition, GC may also signal through protein-protein interactions between receptors and other important transcription factors, including the nuclear factor-κB (NF-κB), the activator protein-1 (AP-1), and the signal transducers and activators of transcription (STATs). However, perhaps even more importantly, GC exert also rapid, nongenomic actions, mediated by membrane-bound MRs and GRs that trigger the activation of kinase signal transduction pathways (8, 52, 54–57, 69). Membrane-bound MRs and GRs show lower GC affinity than intracellular receptors and are increasingly occupied only through higher cortisol concentrations, thus mainly playing a crucial role in translation of rapid GC pulses in the initial phase of HPA axis activation (70–72).

## The Human Circadian System

Circadian molecular oscillations are independently generated in virtually every cell of living organisms, thus influencing molecular biological processes over the course of the day. However, it is the orchestration of these innumerable, diverging and tissue-specific peripheral oscillations into a main rhythmic symphony that is of vital importance for the promotion of homeostasis in higher organisms. The CS represents an extensive network of time-keeping mechanisms that creates and maintains this cellular and systemic rhythmicity, through temporal organization and coordination of many physiological and transcriptional oscillating processes throughout several structural levels in the organism (17, 18). In order to stay adjusted to the geophysical time, the CS receives continuously input by behavioral, hormonal, and environmental signals, a process called entrainment.

The mammalian CS is organized in a hierarchical manner with a central, pacemaking, and light-sensitive “master clock” in the CNS and a peripheral, subordinated multioscillator component (“slave clocks”), showing both top-down and bottom-up organization based on positive and negative endocrine, autonomic, and transcriptional regulatory feedback loops (15, 73–75). The CS has three main functions as (a) pacemaker through intrinsic and self-sustainable rhythm generation, (b) internal *Zeitgeber* (*germ*. time-giver) with a distinct rhythm output for peripheral synchronization, and (c) *Zeitnehmer* (*germ*. time-taker) continuously receiving time-shifting signals from external/secondary *Zeitgebers* (e.g, nutrition, light, sleep, social activity) for proper time entrainment of the intrinsic period to the environmental cycle (76).

### The Central and Peripheral Circadian System

The central mammalian CS includes specialized signal transduction mechanisms in the retina, the retinohypothalamic tract (RHT), the suprachiasmatic nucleus (SCN), the superior cervical ganglia, the pineal gland (PGL), the thalamic intergeniculate leaflet (IGL), and the raphe nuclei (18, 77, 78) (cf. **Figures 1** and **2**). The SCN is a bilateral paired structure with high cell density, consisting of 50,000 neurons (in humans) displaying a synchronised rhythmic metabolic and electrical activity, and is located in the anterior hypothalamus directly over of the optic chiasm, next to the third ventricle. The SCN is the integrative “master clock” of the organism, by integrating its distinct primary pacemaker activity through intrinsic neural firing and all received environmental *Zeitgeber* cues to a main circadian rhythm (17, 18, 79–81). The most important *Zeitgeber* is light. The SCN receives photic input (photoentrainment) from the rod/cone photoreceptors and particularly from other nonimage-forming photosensitive cells in the retina, the intrinsically photosensitive retinal ganglion cells (ipRGCs) (77). These melanopsin-containing cells have been shown to be sensitive to light wavelengths (460–480 nm, i.e. blue light) different from the classical visual system (i.e., rod and cone cells) and they react slowly and tonically to luminance changes (77, 82–87). The photic input transmitted from the ipRGC through the retinohypothalamic tract to the SCN (88) and from there to the upper part of the thoracic spinal cord, the superior cervical ganglia and the PGL gland (89). The NPY-containing pathway from the IGL and the serotonergic pathway from the median raphe represent the two other main afferent projections to the SCN (78). Taken together, anatomical routes directly involved with the SCN are numerous, with up to 15 efferent and 35 afferent projections (78).

The peripheral, subordinated multioscillator component of the CS (“slave clocks”) show a similar, tissue-specific, self-sustained, and cell-autonomous rhythm generation machinery, regulating several functions of their residing tissues, with one essential difference to the central CS: These peripheral “slave clocks” do not exchange phase information and must therefore kept synchronized by the main integrative SCN rhythm *via* different pathways (16), which leads to a 4-h optimal phase synchronization delay of peripheral with respect to the central CS rhythm (90). This synchrony gets mostly lost without an input from the SCN (91), although other *Zeitgebers*, such as nutrient, temperature, and social cues, can also entrain peripheral clocks (92).

### The Molecular Clockwork

In the past decades, mounting evidence has evolved our understanding from the first discovered clock gene (Period or PER) conserved from fruit flies to humans (93) to a complex molecular clockwork generated at the cellular level by molecular oscillators in all nucleus-containing cells of an organism (15, 74, 94). The intrinsic circadian rhythmicity of the biological clock is based on a core set of clock genes intertwined with an autoregulatory, delayed, interlocking transcriptional/translational feedback (TTFL) loop machinery, coupled to several auxiliary mechanisms and leading to mutual transcriptional activation and repression, ultimately maintaining an approximately 24-h oscillation, thus, reinforcing robustness and stability of the clock (14, 15, 74, 94–97).

**Figure 3:**
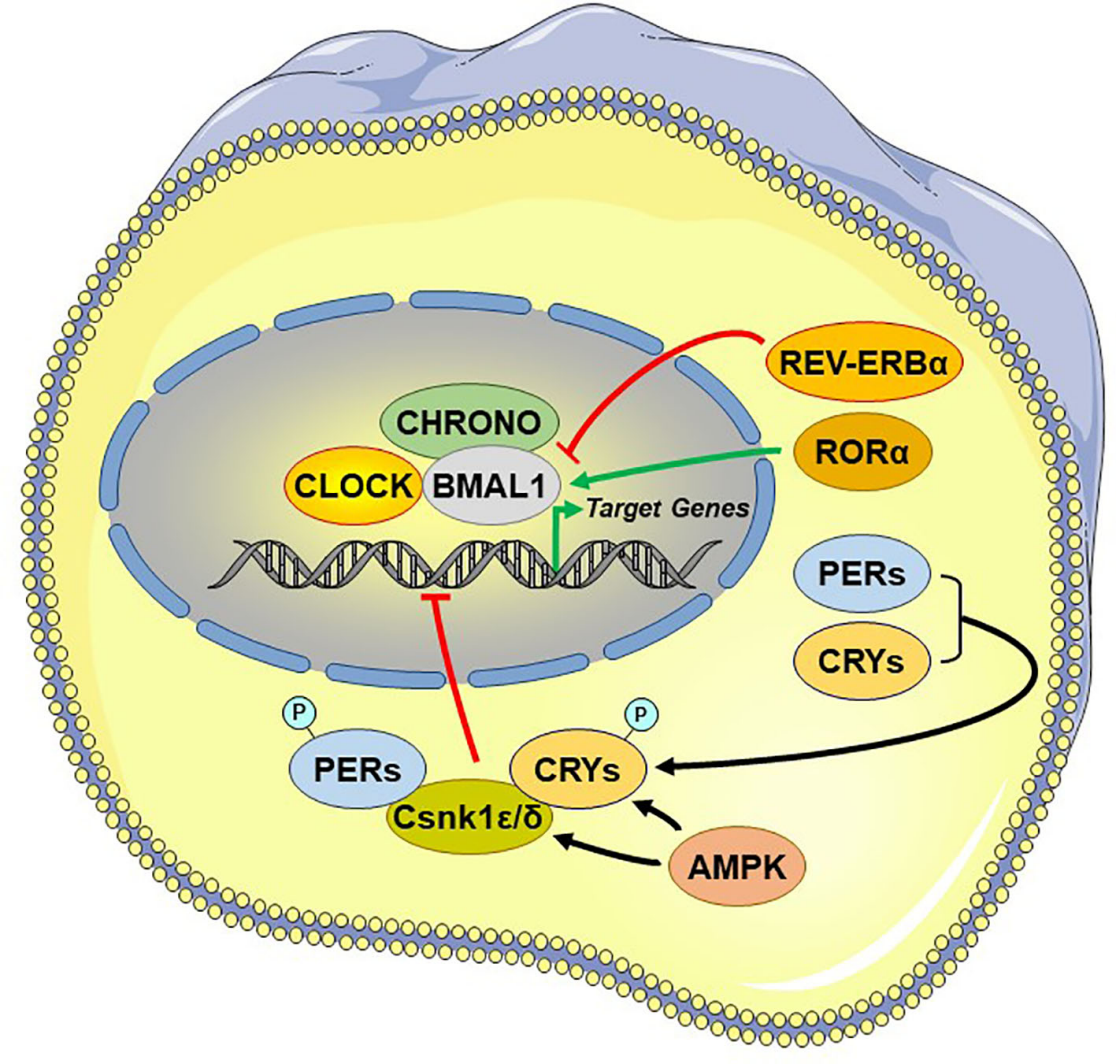
Principal and auxiliary transcriptional/translational feedback loops of the circadian system. AMPK, adenosine monophosphate (AMP)-activated protein kinase; BMAL1, brain-muscle-arnt-like protein 1; CHRONO, ChIP-derived repressor of network oscillator; CLOCK, circadian locomotor output cycle kaput; CRYs: cryptochromes; Csnk1ϵ/δ, casein kinase 1ϵ/δ; P, phosphate residue on the phosphorylated molecules; PERs, periods; RORα, retinoic acid receptor-related orphan nuclear receptor α. REV-ERBα, reverse viral erythroblastosis oncogene product alpha.

Central among the core TTFL are the transcriptional activator “circadian locomotor output cycle kaput” (CLOCK), its heterodimer partner “brain-muscle-ARNT-like protein 1” (BMAL1), and the essential negative regulating circadian genes “Period 1, 2, and 3” (PER1-3) and “Cryptochrome 1 and 2” (CRY1/2) (98). The activated CLOCK/BMAL1 heterodimer binds to the enhancer box (E-box) response elements located in the promoter region and stimulates the transcription of PER1-3 and CRY1/2 at circadian dawn (circadian time 0, CT0). PER1-3 and CRY1/2 mRNA gets translated into proteins, which accumulate by the end of the circadian day (CT12). Over the course of the circadian night (CT12–CT0), inhibitory complexes of PER1-3 and CRY1/2 with the caseine kinase 1ϵ and δ, are phosphorylated and translocate from the cytoplasm into the nucleus and repress the transcriptional activity of the CLOCK/BMAL1 in the SCN, shutting down PER1-3/CRY1/2 transcription (99). After degradation of nuclear PER1-3/CRY1/2 complexes the next morning (CT0), the inhibition on CLOCK/BMAL1 transcriptional activity is released and thereby a new cycle starts over after approximately 24 h (79) [cf. **Figure 3**]. During the circadian day, PER1-3 and CRY1/2 transcription is high in the SCN, leading also to high SCN electrical activity. Besides this core negative feedback loop, there are also auxiliary feedback loops that stabilize the transcriptional activity of the core regulatory loop (94, 100–102). CLOCK/BMAL1 upregulates, for example, the expression of other clock-related proteins, such as the reverse viral erythroblastosis oncogene product α and β (REV-ERBα/β) and the retinoic acid receptor-related orphan receptor α (RORα), which, in turn, regulate BMAL1 expression. Genetic polymorphisms in these clock genes are responsible for a great distribution of entrained phases (chronotypes) between individuals, ranging from “larks” to “owls,” with most individuals falling between these extremes (103).

The transcription factors of both principal and auxiliary TTFLs can modulate the expression levels of many clock-responsive genes in various tissues, influencing a broad spectrum of physiologic functions, such as hormonal fluctuations, sleep/wakefulness, feeding, immune activity, thermoregulation, energy household, and glucose metabolism (14). These regulatory loops, receive adjustive input from related influencing systems. Besides the strongest circadian entrainment by light, other biological cues, such as nutrition and temperature, can also influence the activity of the clock system. For example, peripheral clocks can be influenced by food-related signals through adenosine monophosphate-activated protein kinase (AMPK), a tissue sensor and master regulator of energy balance, which phosphorylates Per1-3 and Cry1/2 leading to their degradation (104, 105). Similarly, temperature decrease can represent a strong circadian cue, as the cold-inducible RNA-binding protein CRBP accumulates under lower body temperature in peripheral clocks (but not in the SCN) and influences circadian gene expression (106).

### Circadian System Interconnections and Effector Pathways

The superior robustness and resilience of the distinct intrinsic activity rhythm of the SCN is mainly preserved by the synchronization of SCN neurons through intercellular coupling to its neighbour cells in an action-potential-dependent manner (107). There are different kinds of SCN neurons containing different neuropeptides, such as arginin-vasopressin (AVP), vasoactive intestinal peptide (VIP), γ-amino-butyric-acid (GABA), glutamate, gastrin-releasing peptide, and somatostatin. This large variety of neuropeptides within the SCN ensures a rich diversity in signalling properties to effector targets (108). According to its neurocircuit topology, the SCN can be functionally divided into two subregions. The dorsomedial shell region primarily produces AVP and gets mainly innervated by the hypothalamus, while the ventrolateral core region primarily produces VIP and receives photic input [cf. **Figure 1**]. SCN output projections target many different brain regions and modulate the activity of downstream neurohumoral pathways in a rhythmic manner, herewith influencing a plethora of physiological processes (14, 16, 109). The most important effector targets of the SCN include: (i) hypothalamic centers associated with activity, temperature, and sleep regulation, such as the subparaventricular area (subPVN) and the dorsomedial nucleus of the hypothalamus (DMH) (110), (ii) preautonomic hypothalamic neurons, affecting vagal and sympathetic autonomic centers in brain stem and spinal cord and, thus, exerting circadian control throughout the body *via* ANS activity (80), and (iii) neuroendocrine hypothalamic centers responsible for hormone secretion (e.g., CRH synthesizing PVN parvocellular neurons) [cf. **Figures 2** and **5**]. The PVN is a significant integrating center for energy homeostasis and distribution center of circadian rhythmicity to the body, as its parvocellular neurons project to the median eminence to control the release of ACTH and thyroid-stimulating hormone (TSH) in the anterior pituitary (i.e., hypothalamic-pituitary-adrenal axis, HPA axis; hypothalamic-pituitary-thyroid axis, HPT axis), and also innervates the sympathetic limb of the ANS (22).

**Figure 4:**
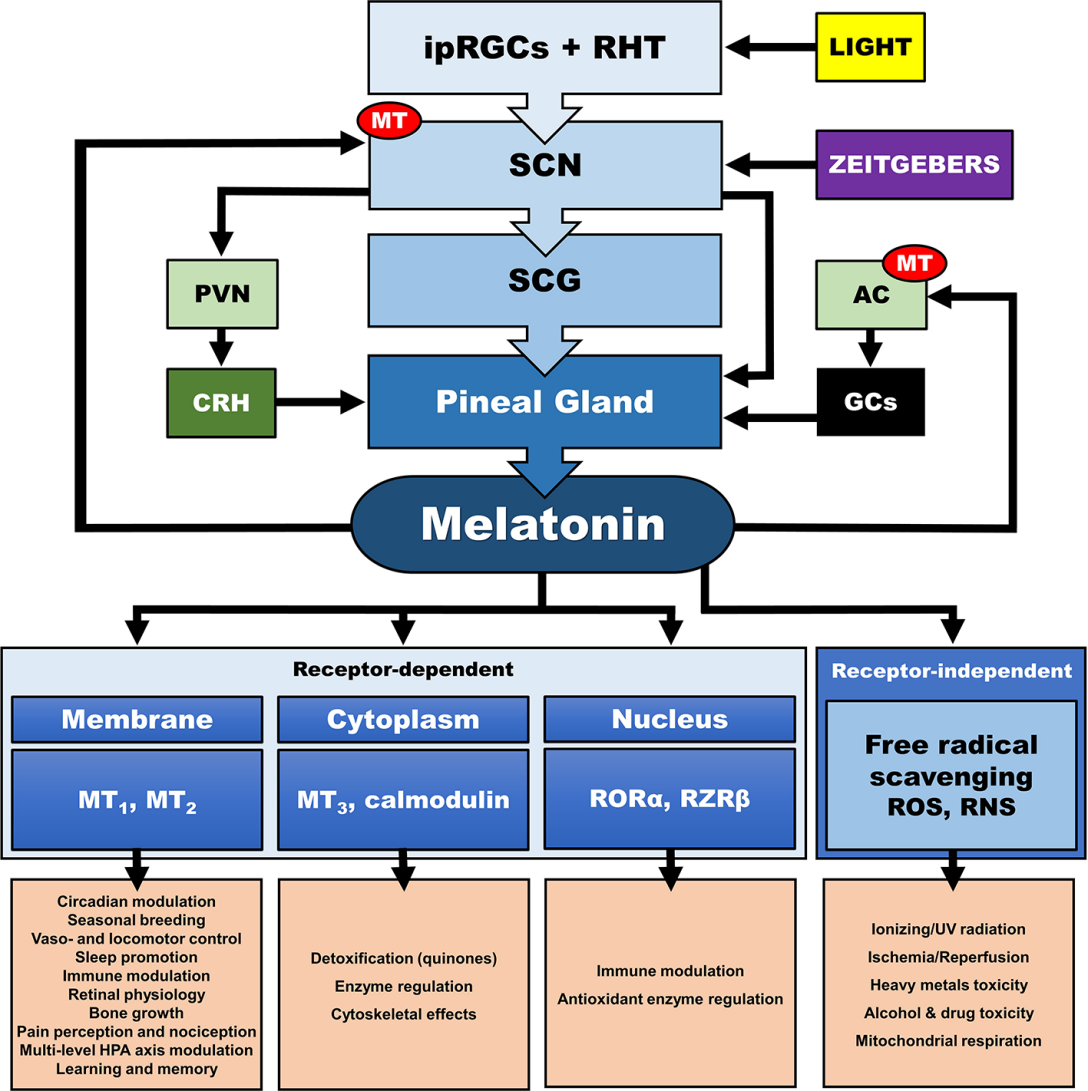
Multilevel interactions between the circadian system and the hypothalamic-pituitary-adrenal (HPA) axis. AC, adrenal cortex; CRH, corticotropin releasing hormone; GCs, glucocorticoids; ipRGC, intrinsically photosensitive retinal ganglion cells; MT, melatonin receptor; PVN, paraventricular nucleus; RHT, retinohypothalamic tract; RNS, reactive nitrogen species; RORα, retinoic acid receptor-related orphan receptor α; ROS, radical oxygen species; RZRβ, retinoid acid receptor related Z receptor β; SCN, suprachiasmatic nucleus; SCG, superior cervical ganlia.

In addition, the central CS, exerts its synchronizing effects also through humoral (i.e., endocrine/paracrine) signals. The main effector of the central CS and essential synchronizing hormone is pineal melatonin (MLT) (111–114), whose secretion is strictly modulated by the SCN and sympathetic fibers originating from the superior cervical ganglia (112, 113, 115–117). Reversely, MLT is a direct modulator of the SCN neuron electrical activity (118, 119), as SCN expresses a high number of MLT receptors (MT) (120), while it also interacts with “clock” gene TTFLs in the SCN, and so modulates circadian rhythms and adjustment to environmental photoperiod changes (121). MLT modulates central and peripheral oscillators and related secondary molecular pathways mainly by cell-specific control through G-protein-coupled MLT membrane receptors MT_1_ and MT_2_ (118) and GABAergic mechanisms (119, 122, 123) [cf. **Figure 4**]. MT is broadly distributed in the body and are vital for immunomodulation, endocrine, reproductive and cardiovascular regulation, cancerogenesis, and aging. Additionally, MLT interacts with cytoplasmic factors (i.e. quinone-reductase-II/MT_3_ receptors, calmodulin) and nuclear receptors (i.e. retinoid acid receptor related orphan and Z receptors, ROR, RZR), while numerous other actions of MLT are receptor independent (e.g., radical scavenging) (114, 124–128). MLT concentration reaches high levels at night (plasma peak between 0200 h and 0400 h), overlapping with decreases in core body temperature, alertness, and performance (111, 113). The sharp elevation of nocturnal cerebrospinal fluid (CSF) MLT exerts substantial protective effects and is responsible for nocturnal tissue recovery after the daily free radical brain damage due to high oxygen utilization (129). These multifaceted chronobiotic regulatory actions have led to the recognition of MLT as one of the most pleiotropic biological signals in photoperiodic species (114, 130). On the other hand, it is important to note that the majority of laboratory mouse strains do not produce melatonin and thus challenge the importance of MLT in related animal findings (131).

Finally, sleep acts restorative in concert with the CS, but also independently, towards optimizing the internal temporal order (132). Sleep propensity and sleep stage timing, regulated through the subPVN and DMH, are bidirectionally associated with circadian gene expression in the SCN (133), but also strongly modulated by MLT levels (119, 134–138).

## Interactions Between the Human Circadian and Stress System

The human CS and SS are closely and bidirectionally interconnected at multiple central and peripheral functional levels (19, 22, 23, 139–148). The circadian properties of the HPA axis are so distinct, that, along with MLT, GCs have been established as a robust measure of CS output activity. Additionally, MLT and GCs can also feedback at various levels and influence the main circadian rhythm themselves. Interestingly, the phase angle between CORT and MLT onset, the two major hormonal output signals of the CS and the HPA axis, has been identified as a potential useful biomarker in human stress-related research (149).

### Influence of the CS on SS Activity and Reactivity

The HPA axis shows distinct circadian activity at rest with a robust diurnal oscillation of circulating GCs (i.e., cortisol, CORT) concentrations, rapidly rising in the middle of the biological night and peaking in the early morning, reaching their nadir before the habitual inactive phase onset (19, 141, 142, 150). SCN ablation completely abolishes the GC circadian rhythm, suggesting that HPA axis activity is driven by the central CS (151). In addition, the CS has a major influence on the ANS. Major human cardiovascular markers, such as heart rate, blood pressure, baroreflex, heart rate variability (vagal measure), plasma epinephrine, and norepinephrine levels (sympathetic measure) and their response to stressors exhibit robust circadian variations with a distinct peak of sympathetic activity and nadir of parasympathetic activity in the morning hours (152–157). By doing so, the HPA axis and SNS activity are believed to prepare the organism for the higher energetic demand associated with typical external and internal stressors of the waking phase (24).

#### Neurohumoral Interactions

**Figure 5:**
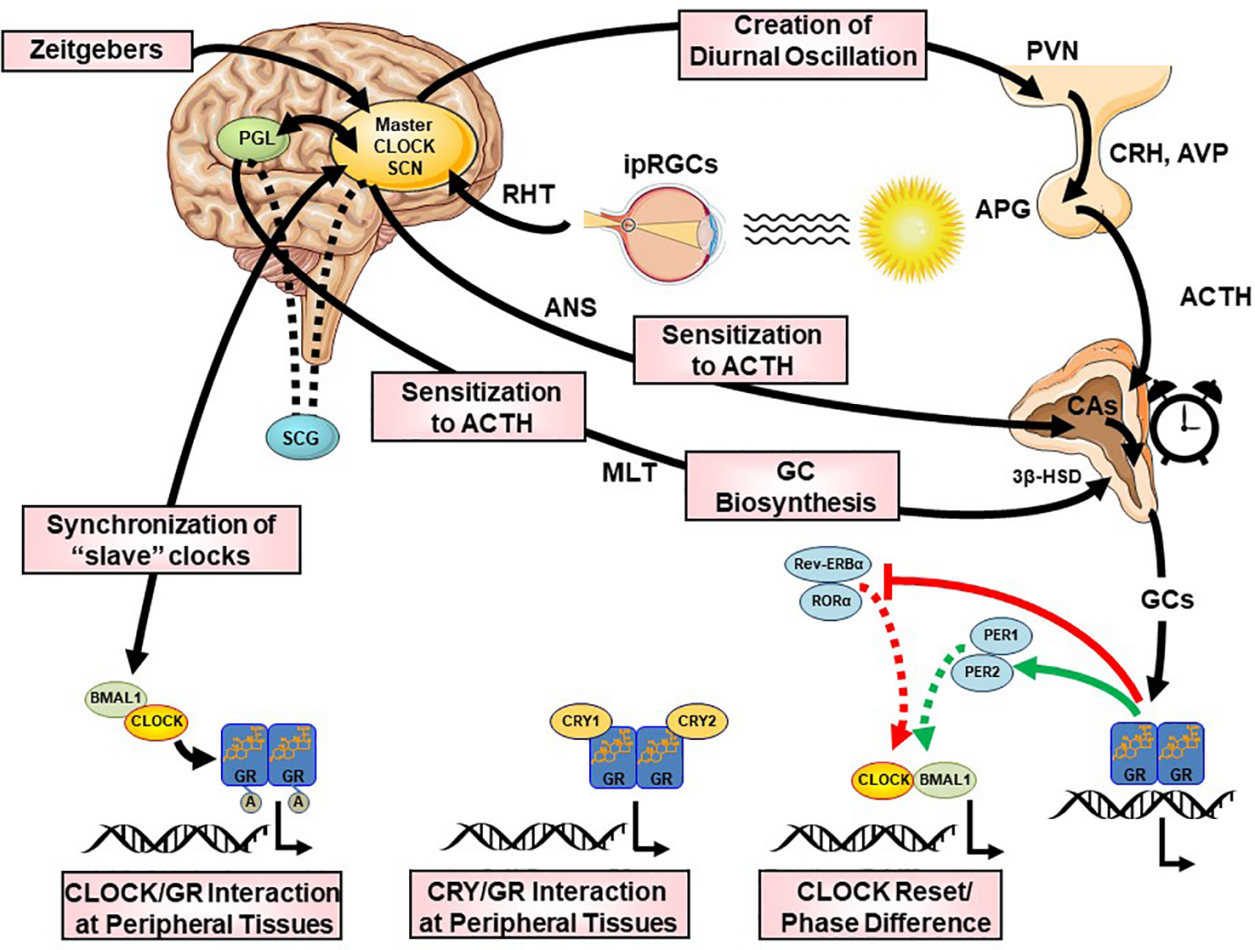
Multilevel interactions between the circadian system and the hypothalamic-pituitary-adrenal (HPA) axis. ACTH, adrenocorticotropic hormone; APG, anterior pituitary gland; AVP, arginine vasopressin; BMAL1, brain-muscle-arnt-like protein 1; CA, catecholamines; CLOCK, circadian locomotor output cycle kaput; CRH, corticotropin releasing hormone; CRYs, cryptochromes; HSD, hydroxysteroid dehydrogenase; ipRGC, intrinsically photosensitive retinal ganglion cells; GCs, glucocorticoids; GR, glucocorticoid receptor; PERs, periods; PVN, paraventricular nucleus; REV-ERBα, reverse viral erythroblastosis oncogene product alpha; RHT, retinohypothalamic tract; RORα, retinoic acid receptor-related orphan nuclear receptor alpha SCN, suprachiasmatic nucleus.

The CS orchestrates the circadian activity and reactivity of the HPA axis through both hormonal and neuronal pathways. There are three main pathways of CS influence on the HPA axis: (i) direct SCN influence on HPA axis at the hypothalamic level, (ii) SCN innervation of the adrenal glands through indirect, multisynaptic autonomic innervation, and (iii) peripheral rhythms of local adrenal clocks, all three involved in the steroidogenic pathway and the ACTH-dependent transduction cascade in the zona glomerulosa and zona fasciculata of the adrenal gland (158) [cf. **Figures 2** and **5**]. The first pathway includes direct and indirect (through subPVN and DMH) neuronal projections of the SCN to CRH/AVP containing neurons of the medial parvocellular PVN modulating the circadian secretion of CRH and AVP (108, 146, 159, 160). Through the second pathway, the SCN transmits photic information *via* multisynaptic autonomic innervation (i.e., preganglionic intermediolateral projections to the spinal cord and splanchnic nerve innervation) to the adrenal medulla and from there through catecholamines to the cortex (161), thus both modulating the diurnal ACTH sensitivity of the adrenal cortex and stimulating the GC circadian release in light exposure conditions through an HPA axis-independent manner of direct interaction with the own peripheral rhythm of the adrenal gland (i.e., PER1 and StAR gene expression) (7, 140, 162–169). Interestingly, SCN neurons display connections to SNS and PNS, indicating that the SCN is not only essential for the physiologic autonomic diurnal fluctuations seen in humans (153, 155, 157), but also involved in both activation and deactivation of neuronal innervation of the adrenal in a circadian circle (80). The intrinsic circadian rhythm of adrenal glands in metabolic activity and GC release even in culture has been shown very early in literature (170), while clock genes expression was repeatedly reported in the following years (140, 164, 165, 171, 172). However, additional adrenal-intrinsic mechanisms depending on systemic cues, such as food-entrainable oscillators of the gland, could influence the diurnal rhythms of GC secretion (173, 174). Another very important mechanism for shaping the GC circadian rhythm is their own systemic levels, exerting a negative feedback regulation of ACTH release (175). The sensitivity of this feedback mechanism is highest during the trough point of the circadian glucocorticoid rhythm depending only MR at this time, while both MR and GR are involved at the GC peak-point lowest sensitivity (175). Finally, MLT, apart from its direct modulating effect on the SCN (176), has been also shown to directly influence GC production and release by the adrenal gland, as well as acetylation rhythms of GR, GR translocation to the nuclei and transcriptional activity (125, 172, 177, 178). MLT has been found to prevent adrenal response to ACTH (177, 179) and directly inhibit CORT production through MT1 adrenal receptor activation, possibly through their action on the Type II 3β-HSD (3β-Hydroxysteroid-dehydrogenase/Δ^5-4^ isomerase) enzyme activity, which catalyzes the biosynthesis of hormonal steroids through the oxidation and isomerization of Δ^5^-3β-hydroxysteroid precursors to Δ^4^-ketosteroids (180). Taken together, this illustrates the multilevel circadian “gating” control on the physiological GC secretion rhythm through SCN, HPA axis and ANS activity, GC and MLT levels, feeding and the robust intrinsic rhythm of the adrenal gland itself, involving clock gene expression in the metabolism and secretion of GCs (80, 140, 141).

In addition, MLT acts directly through MT_1_/MT_2_ on the electrical activity in SCN neurons (118, 119) and interacts with the “clock” gene (PER1/2, CRY1/2, CLOCK, BMAL1, etc.) proteasome TTFL in the SCN, thus being crucial for circadian entrainment in photoperiodic species (121).

#### Molecular Interactions

The neurohumoral interactions between CS and SS described above, have further molecular underpinnings at the cellular level, where the GR plays a fundamental role. For example, findings suggest that the CLOCK/BMAL1 heterodimer behaves as a reverse-phase negative regulator of hGRα in the periphery, antagonizing the physiologic actions of diurnally fluctuating GCs. Through a region enclosed in the C-terminal part of the CLOCK protein, CLOCK/BMAL1 physically interacts with the ligand-binding domain of hGRα and acetylates the hGRα at multiple lysine residues, thereby reducing GR’s affinity to its cognate glucocorticoid response elements (GREs) and, thus, leading to decreased hGRα-induced transcriptional activity of glucocorticoid-responsive genes (144, 181–184). GR transactivational activity fluctuates in a circadian fashion and in reverse phase with CLOCK/BMAL1 mRNA expression (182) and leads to a higher hGRα acetylation and decreased tissue glucocorticoid sensitivity in the morning, mirroring the circadian pattern of serum CORT concentrations (183). In addition, a CLOCK-mediated posttranslational modification of hGRα is involved with the nuclear localization signal 1 (NL1), altering the cytoplasm-to-nucleus translocation of the receptor following ligand-induced activation, and indicates that the hGRα acetylation by CLOCK is linked to several molecular mechanisms (182). Moreover, Lamia and collaborators demonstrated that CRY1/2 interacted with the carboxyterminal domain of hGRα, thereby reducing the DNA-binding of the receptor and its transcriptional activity (185). Interestingly, the effect of a specific clock gene deletion on circulating GCs seems to depend on the specific TTFL missing member, suggesting that alteration of the positive or negative limb of the core clock feedback loop may have opposing effects on stress regulation. Accordingly, BMAL1 (TTFL positive limb gene) deletion leads to low adrenal ACTH sensitivity throughout the circadian circle, supporting constant low GC levels and insensitivity to acute stress (186). Genetic deletion of CRY1/2 (TTFL negative limb genes) leads to nonoscillating and elevated GC levels due to impaired feedback inhibition (185, 187). In contradistinction, the PER1/CRY1 complex reduces the maximal GR transactivation but not the efficacy of the receptor (184). Furthermore, CHRONO (ChIP-derived repressor of network oscillator), which is encoded by a BMAL-target gene, interacted with BMAL1, CRY2 and DEC2 and recruited the histone deacetylase 1 (HDAC1) to the transcriptional machinery, ultimately repressing the principal transcriptional loop (188). CHRONO is also able to acetylate the hinge region lysine cluster of GR, reducing its DNA-binding and thus indicating that this protein might play a fundamental role in the interaction of the CS with the SS (182, 188, 189). More recent *in vitro* and *in vivo* studies also showed that REVERBa, in interaction with heat-shock-protein (HSP) 90, influences the stability and nuclear localization of GR in the liver and provides another link between the CS, metabolism and glucocorticoid actions (190, 191). In addition, transcriptional cofactors of nuclear receptors (e.g., PGC1a) has recently been also implicated in circadian clock function (192), while interacting with the GR (193). Similarly, HSP, forming a dynamic complex with the GR in the cytoplasm (i.e., before GC binding and nuclear translocation), also display a circadian regulation through systemic circadian temperature changes, thus contributing to clock entrainment in peripheral tissues (194, 195). Finally, FKBP5, a chaperone protein of particular interest involved in directing activated GRs to the nucleus and implicated in a number of stress related psychiatric disorders, is also rhythmically expressed in most tissues (196), suggesting its involvement in circadian gating of GC signals.

### Influence of the SS on the Central and Peripheral CS

Apart from the influence on many important biological processes, the rhythmic oscillations of the SS activity and especially the HPA axis and GC rhythmicity exert a vital synchronizing effect on the central and peripheral CS activity (19, 23, 92). GCs, through binding to the hGRα, can efficiently reset the activity of peripheral clocks (197–199), while they spare the SCN, which maintains its master intrinsic circadian rhythm, as it does not express GRs (158, 197). The attenuation of the peripheral clocks by the phase-shifting effects of the GCs is then normally restored by the influence of the SCN. However, the SS has to directly influence the SCN through an alternative pathway, as both stress exposure and exogenous GC application enhances AVP and VIP mRNA expression and release in the SCN (200, 201), while acute stress exposure also leads to an upregulation of Per1 and Per2 protein expression in the SCN (202). For example, CORT and CRH are suggested to directly modulate PGL activity and stimulate MLT synthesis, interfering in the daily adjustment of the light/dark cycle (179, 203–206). In addition, GCs play an important role in the adjustment of nutrition-related uncoupling between the central and peripheral CS, as their high secretion after feeding slows down the circadian uncoupling and restores proper phasing (173, 207). GCs are, thus, not just a downstreal hormonal output of central and peripheral clocks, but can also influence the CS itself and interact with other clock outputs toward a harmonious circadian regulation (141, 197), adding another interaction level between the stress and the circadian clock system. Alteration of the GC rhythm (e.g., through exogenous GC administration) can, thus, attenuate the central and peripheral circadian activity and vice versa (167, 208). Taken together, the SS through its effectors efficiently adjusts the circadian rhythm-linked output pathways of the body to properly respond to stressors, providing resistance to stress challenges in order to evade uncordinated circadian shifts (23).

#### Molecular Interactions

Diurnally circulating GCs vitally contribute to the development of the CS activity by adjusting the phase of peripheral oscillators (19, 148). GCs synchronizing effects mainly involve GR-related phase shifting of peripheral circadian expression of several clock-related genes (197, 209–216). All peripheral clocks express GR, which translocate into the nucleus after activation and modulate transcriptional activity of several clock genes (e.g., PER1/2) and transrepressing genes expressing transcription factors of the auxiliary TTFL (e.g., Rev-ERBα, RORα) through binding to functional GREs in their promoter region (217–222). PER1 contains GRs in its regulatory sequences, while GRs influence the expression of PER2 through binding to an intronic domain (218). GCs lead herewith to upregulation of these genes, causing a phase delay of peripheral clocks with respect to the SCN master clock (218). A genetically, functionally (e.g., adrenalectomy) or pharmacologically (i.e. externally administered corticosteroids) attenuated GC diurnal rhythm has been shown to be associated with abolished or shifted circadian clock gene (e.g., PER1/2) expression in several peripheral tissues (e.g., liver, preadipocytes, kidney, bronchial epithelial cells, pancreas, bone tissue, cornea, fibroblasts cardiac muscle tissue), despite the presence of an intact molecular oscillator (167, 197, 208, 209, 215, 216, 223–226). Even externally applied corticosteroids can entrain molecular oscillation in peripheral clocks (215, 227) and have been shown, for example, to speed up or slow down adaptation to a new light-dark schedule after jetlag-induced circadian desynchrony (198).

However, rhythmic GC signaling is also required for periodic clock gene expression in certain brain regions outside the SCN, suggesting an important role of the adrenal rhythm also for higher brain functions in key stress-system-related regions (228). Indeed, GR-mediated GC signaling is, for example, fundamental for the rhythmic expression of PER2 in the amygdala (213, 229), while adrenalectomy is shown to supress and extended GC exposure to increase PER gene expression in the PVN, bed nucleus of stria terminalis (BNST) and other limbic areas (219, 228, 230–232). GC-dependent circadian gene expression could even be indirectly involved in a GC feedback pathway to the SCN (233). For example, serotonergic projections of the raphe nucleus to the SCN involved in light entrainment (234) show a GC-dependent circadian transcription of tryptophan hydroxylase-2 (TH-2), an enzyme involved in serotonin synthesis (235).

Finally, the SAM/ANS constitutes another pathway in stress-induced peripheral circadian entrainment. Administration of adrenaline or noradrenaline has been shown to induce PER1/2 expression through the cAMP response element-binding protein (CREB) signalling pathway (236–238). Furthermore, GR-related GC effects and clock machinery also interact through a modulation of catecholamine biosynthesis and degradation, thus influencing time-of-day-dependent stress responses and further reinforcing the interaction between the CS and the SS (94, 239, 240). Catecholamine biosynthesis is both GC- and clock-regulated, as TH (i.e., the main synthesis pacemaker enzyme) is repressed by Rev-ERB*α* (241) and induced by the GR-activated the nuclear orphan receptor NURR1 (NR4A2) (242). Similarly, catecholamine degradation depends on the CLOCK/BMAL1-activated monoamine oxidase I (MAO-A) and the GR-regulated catechol-O-methyltransferase (COMT) (243).

Taken together, GC rhythms exert an accompanying circadian signal which consitutes an additional level of security to ensure proper circadian signalling input to the cell cycle oscillating machinery, while, on the other hand, peripheral clocks might gate this GR-specific input.

## Stress and Circadian Misalignment

### Chronodisruption and Sleep Dysregulation

The human CS enables the nyctohemeral organization and coordination of many temporal physiologic processes promoting homeostasis and environmental adaptation (18). A misalignment of the human circadian rhythm is associated with a critical loss of this harmonious biological timed order at different organizational levels, which is defined as *chronodisruption* (244–246). Chronodisruption-related cacostatic load with short- and long-term pathophysiologic and epigenetic consequences (245–247) can lead to a wide range of biological consequences in the organism (246, 248–255). Chronodisruption may gradually change the fundamental properties of brain systems regulating neuroendocrine, immune, and autonomic function and denotes a breakdown of appropriate biobehavioral adaptations to challenges with increased stress sensitivity and vulnerability to stress-related disorders (20, 256, 257).

In human research, chronodisruption has been tightly associated with sleep deprivation/dysregulation (SD) or phase shifting (i.e., jet-lag, swift-workers) (81, 132, 258). Sleep acts in concert with the central CS, but also independently towards an optimal internal temporal order (132). Specific sleep stages are closely related with specific clock gene expression in the SCN and are tightly ruled by the CS (81, 132, 258). SD has been associated with circadian-related gene expression alterations in humans (259–262). In addition, SD also relates to various HPA axis dysregulations (e.g., flattened CORT rhythm amplitude, blunted CORT awakening response (CAR), increased but also decreased diurnal CORT levels, higher CRH levels) and altered endocrine stress reactivity (e.g., attenuated pituitary ACTH reactivity, increased adrenocortical ACTH sensitivity) (257, 263–269), as well as to altered autonomic regulation with increased sympatho-adrenal and reduced vagal activity and blunted cardiovascular autonomic rhythmicity and autonomic reactivity (257, 270–273). Accordingly, chronodisruption in humans has been associated with increased risk for cardiovascular morbidity, metabolic consequences, inflammation, immune dysregulation, psychiatric disorders and even elevated cancer risk (226, 240, 274–280). Interestingly, even circadian gene polymorphisms have been associated with some similar consequences (281, 282).

### Stress and Chronodisruption

In addition to other crucial circadian cues that can dysregulate circadian rhythms (e.g., SD, nutrition, light), stress can also lead to acute/reversible or sustained chronodisruption. Normally, after exposure to stressors, the SS can transiently override the CS creating a transient uncoupling of the central and peripheral circadian rhythm, through a hGR-related phase shift of peripheral clock-related genes (182, 197, 207, 212, 217, 221, 283). Thereby, the SCN is only indirectly influenced (198) and is, thus, able to maintain its master rhythm and restore its initial main phase to the periphery after stress termination (283, 284). Indeed, subacute stressors have been experimentally shown to have only transient impact on SCN-regulated rhythms in animal research (285, 286). However, the stability of the SCN clock appears to fade away after extensive acute or chronic physical, psychological, inflammatory, or metabolic stress (25). For example, in a study comparing single versus chronic social defeat across two weeks, single stress exposure advanced only the adrenal peripheral clock, while chronic stress also clocks in the CNS (287). Animal research provides additional evidence that chronic mild stress disrupts the regulated gene expression of several clock genes in several peripheral (287, 288), but also CNS tissues, including the hippocampus, amygdala, PFC (202, 286, 289, 290) and the SCN (283, 284, 291, 292). Chronic stress exposure in mice has been shown to alter the circadian properties of the HPA axis (293, 294), while extensive physical stress after surgery in humans leads to disturbances in MLT, CORT and core body temperature rhythms (295). In addition, numerous human and animal studies suggest that acute extensive and chronic stress can affect major sleep centers of the brain (202, 205, 288, 289, 293–296) and, thus, influence sleep physiology leading to both immediate and long-lasting sleep disruption (297–299).

### Circadian-Phase-Dependent Stressor Effects

Apart from the physiological circadian activity of the SS, the stress responsiveness also displays diurnal sensitivity changes, probably through differential interference of the SCN to different brain areas (146, 159, 300). For example, acute psychological stress, involving higher brain areas and the limbic system, as well as acute physical external stress (i.e., restraint/immobilization, foot shock, shaking stress) exert the largest stress response during the rest phase (301, 302), when the HPA axis is less responsive, while acute physiological internal stress (i.e., oxidative stress, hypoglycaemia, hemorrhage), relayed to the PVN and brainstem, at the beginning of the activity phase (303, 304), when the HPA axis is most sensitive to stimulation (175). This appears reasonable, as acute physiological internal stress represents a greater threat during the active phase of animals, while acute external physical stressors (e.g., predator attack) during the inactive phase, while animals are asleep.

Interestingly, further experimental findings in animals suggest that repeated external stress exposure (i.e., chronic stress) has a more detrimental effect when applied during the inactive phase, (284, 305–308), while chronic psychosocial stress (i.e. social-defeat paradigm) shows inverse effects and exerts more detrimental effects during the active phase (307, 309) in animal research. These results jointly suggest that the effect of a stressor depends not only on the circadian phase of exposure, but also on the interaction of the circadian phase with the stressor type, as well as with the chronicity of the stressor (25, 310). For example, both physical and psychological stress at the beginning of the light phase leads to a phase advance, while at the beginning of the dark phase to a phase delay of PER2 expression in mice (286).

## Chronodisruption and Traumatic Stress

The stress-related effects on internal rhythms described above have supported a recent research focus on the potential causal role of SD and chronodisruption in the acute pathophysiology and the development of long-term effects of traumatic stress exposure, suggesting that chronodisruption may represent a potential underlying neurobiologic link (311–315). The association between sleep and circadian disruption and psychopathology was first officially noted by Emil Kreapelin in 1883 (316) and has evolved through the years by numerous biological findings (317).

**Figure 6:**
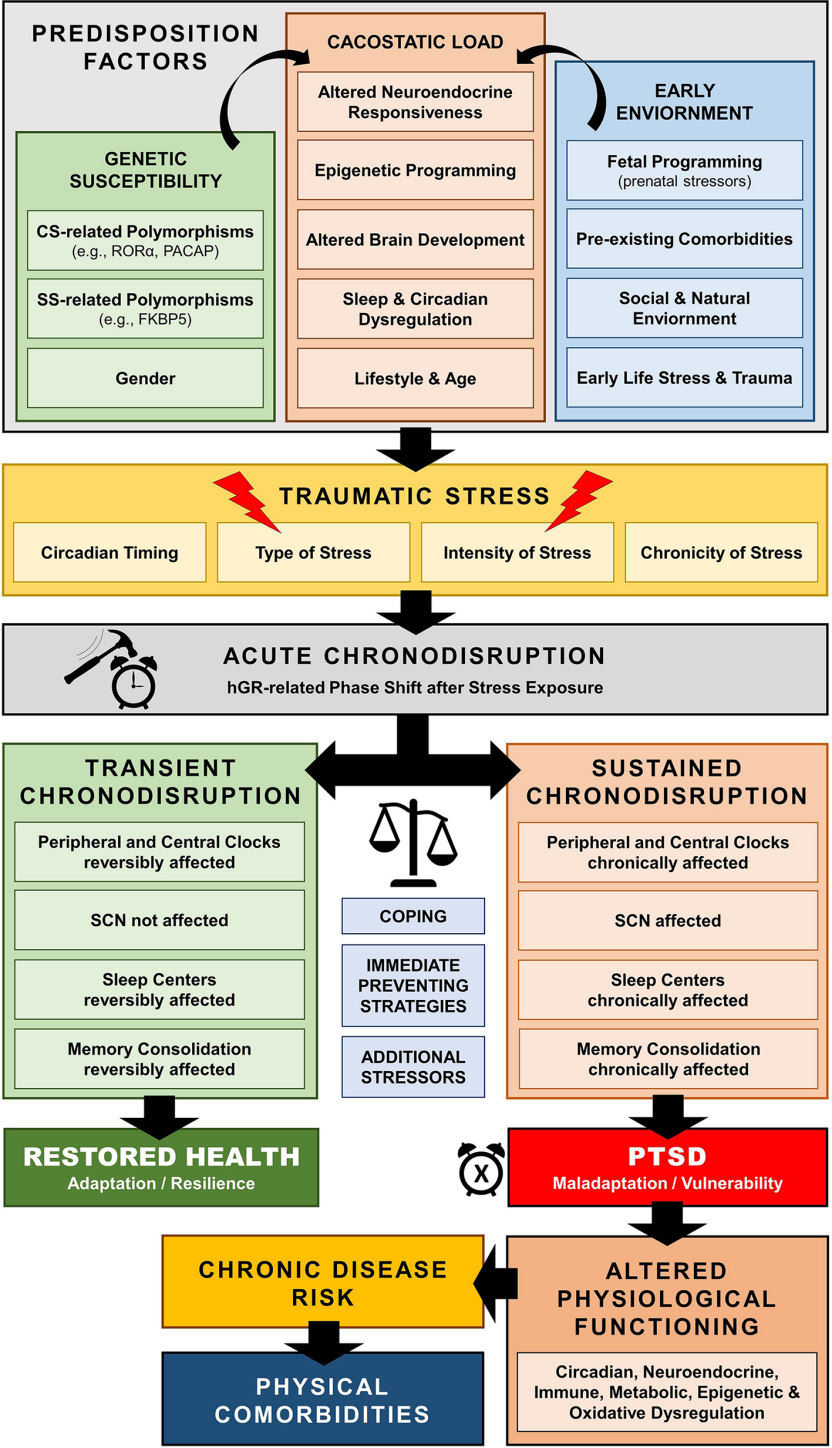
Schematic model of trauma-related chronodisruption as underlying biological pathway leading to posttraumatic stress disorder (PTSD) and PTSD-related comorbidities.

Traumatic stress exposure may cause both immediate and long-lasting SD (297–299), which may represent a central pathway mediating the enduring neurobiological correlates of trauma (297, 311, 312, 318–320) [cf. **Figure 6**]. For example, several human cohort studies have repeatedly suggested that early-life traumatic stress exposure is related to adult SD years later, including global (i.e., insomnia), but also other specific sleep pathologies, such as prolonged sleep onset latency, shortened total sleep time, decreased sleep efficiency, nightmare related distress, increased number of awakenings, sleep apnoea, and higher nocturnal activity (321–332). Such sleep dysregulation could further enhance maladaptive stress regulation and precipitate the neurobiological correlates of traumatic stress through impaired homeodynamic balance, resulting in the extensive symptomatology and comorbidity of trauma-related disorders (314, 333–350).

### Posttraumatic Stress Disorder: When Time Stands Still

Posttraumatic stress disorder (PTSD) is classified in DSM-5 as a trauma- and stress-related disorder following a psychologically distressing event outside the range of usual human experience (351). Evidence of circadian dysregulation in PTSD mostly originates from sleep research findings. According to DSM-5, SD represents prominent clinical feature of the disorder with very high prevalence (312, 320, 351), and is often closely related to severity of PTSD psychopathology (352, 353) and resistant to first-line treatments (354–356).

SD in PTSD is associated with sleep-related arousal dysregulation (357) and include sleep avoidance, insomnia, nightmares, hyperarousal states, sleep terrors and nocturnal anxiety attacks, body-movement and breathing-related sleep disorders (311, 320, 358–362), with increased sympathovagal tone during rapid-eye-movement (REM) sleep, fragmented REM sleep patterns, and reduced REM theta activity (311–313, 318, 363–365). Similar findings have been in animal and human SD studies (366, 367). Interestingly, REM sleep disruption in the immediate aftermath of a trauma (311, 318, 319), as well as sleep impairment prior to traumatic stress exposure could represent risk factors for PTSD development (368, 369). SD prior to trauma have been specifically shown to be associated with a 2.5-fold increased risk of fulfilling PTSD criteria 3 months after a trauma in general population admitted to a hospital or after deployment in active military troops respectively (368, 369). SD after trauma thus represents a rather core than secondary feature of PTSD (297, 311, 312, 318–320, 370) and may be both a precipitating and perpetuating factor of the disorder (371–373).

Besides SD, traumatic stress also affects neural correlates of memory formation (374–376). Memory processing, formation and consolidation are directly influenced by sleep (377–387). Sleep promotes memory consolidation, particularly for emotionally salient information (383), while SD reduces the connectivity between amygdala and PFC (388) thus disrupting memory consolidation (389–393), as repeatedly shown in PTSD.

In addition to SD studies in PTSD, additional CS-related evidence on chronodisruption in PTSD originates from genetic, neuroendocrine, autonomic, and immune findings. For example, genome-wide association studies have also implicated to core circadian genes as PTSD candidate risk genes: pituitary adenylate cyclase-activating polypeptide (PACAP) and retinoid-related orphan receptor alpha (RORA-α) gene. PACAP is involved in phase resetting in response to light (394–396) and RORA-α is rhythmically expressed and regulates BMAL activity (397, 398). Furthermore, as immune system activity tightly follows circadian rhythms imposed by the SCN synchronisation (205, 399–405), our recent first report on the loss of the typical peripheral biphasic rhythm of IL-6 in combat stress exposed individuals (406), is of particular importance.

Further neuroendocrine findings in PTSD repeatedly show increased central CRH levels, altered HPA axis reactivity with enhanced negative feedback inhibition and blunted circadian CORT rhythm and CAR, while some studies—but not all—have shown decreased circulating concentrations of CORT (407–419). Similarly, patients with PTSD exhibit increased autonomic reactivity, elevated central and peripheral norepinephrine concentrations, higher basal heart rate, increased sympathovagal balance, blunted salivary alpha-amylase awakening response and, most importantly, blunted diurnal autonomic differences (341, 417, 420–427), suggesting central neuroautonomic dysregulation leading to higher cardiovascular risk in PTSD (415, 428, 429). In addition, disrupted MLT levels in the first 48 h after traumatic stress exposure were shown to be associated with a higher PTSD development risk (430).

Finally, PTSD has been frequently related to several other comorbidities, such as chronic fatigue syndrome (CFS) (431–434), fibromyalgia (435–439), rheumatoid arthritis (348), which all share a very similar underlying neuroendocrinological profile to PTSD (e.g., hypocortisolism, blunted diurnal CORT rhythm and HPA axis reactivity) (440–445) and have all been repeatedly associated with sustained chronodisruption (446–456).

## Chronotherapeutic Implications for PTSD

Current evidence suggests that SD and CD may have a vital predispositional role in PTSD development (314), while their effective treatment could be associated with substantial improvement of overall PTSD symptomology (312, 457–459). Nevertheless, SD is still often clinically addressed as a secondary symptom in PTSD. Careful assessment and treatment of SD and CD should therefore be an integral part in PTSD management (356, 364, 371–373). Cognitive-behavioral sleep management in PTSD constitutes a widely acceptable and effective treatment option with durable gains and beneficial effects (356, 460–462). In addition, the antihypertensives α-1 adrenoreceptor antagonist prazosin and α-2 adrenoreceptor agonist clonidine, the synthetic cannabinoid receptor 1 and 2 agonist nabilone and the multilemodal antidepressant trazodone (i.e., serotonin-reuptake inhibitor, 5-HT_2A_ receptor agonist, histamine H1 receptor antagonist, α-1 and α-2 adrenoreceptor antagonist) have been all shown to be effective pharmacological approaches for PTSD-related sleep disturbances and trauma-specific nightmares (463–467). Standard pharmacological sleep management in PTSD, however, may treat sleep quantity sufficiently, but often fail to improve daytime functioning and restore CD in PTSD (132, 468). Therefore, development of chronopharmacological interventions that would restore CS-related alterations and herethrough counteract changes in PTSD-related neurocircuitry could represent interesting novel therapeutic strategies (469–472).

### Melatonergic Treatment

Recent experimental findings emphasize on a pleiotropic, but crucial role of MLT in mechanisms of sleep, cognition and memory, metabolism, pain, neuroimmunomodulation, stress endocrinology and physiology, circadian gene expression, oxidative stress, and epigenetics, thus suggesting a potentially beneficiary effect of an add-on melatonergic treatment in PTSD (374, 473). Numerous studies have repeatedly confirmed the efficacy of melatonergic treatment on almost every aspect of sleep disturbance, while preserving a benign side-effect profile and safety in both short- and long-term administration, with no efficacy wear-off, withdrawal effects or dependence risk (119, 474–480). MLT and melatonergic agonists, were found roboustly associated with (i) reduced sleep onset latency and increased sleep propensity, efficiency, quality, and total sleep duration in patients with insomnia, (ii) increased REM sleep percentage and continuity, normalization of sleep patterns, body-movement and breathing-related pathologies and improvements in subjective measures of daytime dysfunction in neuropsychiatric patients and (iii) advanced sleep/wake rhythm phase adjustment and sleep and wake-up propensity in healthy adults (119, 134, 135, 138, 474, 477, 478, 480–486). In addition, MLT is known to adjust and reset amplitude and phase of CNS (e.g., SCN, hippocampus, pituitary pars tuberalis) and peripheral (e.g., adrenal gland) circadian-related gene expression (172, 177, 178, 180, 487–489) and to moderate the circadian regulation of GR function (140, 141, 144, 183, 490). MLT also decreases hypothalamic CRH levels and inhibit the ACTH-stimulated CORT production in the primate and human adrenal gland (172, 177–180, 487–489), thus attenuating the adrenocortical secretory response in acute and chronic stress models (491–494). With respect to the ANS, MLT entrain disrupted autonomic rhythmicity by inhibiting central sympatho-adreno-medullary (SAM) outflow and shifting autonomic balance in favour of vagal activity (154, 495–498). Interestingly, research findings suggest a direct enhancing effect of melatonergic transmission in stimulus processing, memory consolidation, and conditional cued fear extinction, especially under stress (499–502). Finally, immediate melatonergic treatment directly after exposure to stress, normalizes the altered expression of Per 1 and Per 2 genes in hippocampal regions of rats, thus suggesting a possible immediate preventing properties (202). MLT has been shown to protect these hippocampal neurons from oxidative stress, by preventing GC-related toxicity through decrease of receptor translocation to nuclei in models of sleep deprivation and chronic stress (503–506). Taken together, MLT and melatonergic agents could therefore represent a promising adjuvant contribution to the clinical treatment and perhaps prevention of stress-related syndromes and comorbidities in mental disorders in general and PTSD in particular (124, 314, 471, 507–509).

### Other Potential Treatment Possibilities

Further options for a pharmacological or nonpharmacological manipulation of the interplay between CS and SS in order to interfere in the pathophysiology of trauma-related disorders are of theoretical interest and deserve thorough further investigation through preclinical research and clinical confirmation. For example, exogenous application of GCs and GC-analogs in a time-of-day dependent fashion (i.e., as in immune therapy), could contribute to a reset of peripheral clocks (55, 144, 510) or even contribute to PTSD prevention if applied immediately after trauma exposure (511). On the other hand, pharmacological GR-antagonism has been found associated with insomnia symptoms improvement (512) and could also represent a potential approach.

As sleep promotes memory consolidation, particularly for emotionally salient information, sleep deprivation in the beginning of the resting phase directly after traumatic stress exposure may also decrease the risk of PTSD development (513), possibly through reduction of mPFC-amygdala connectivity (388, 390, 392). Furthermore, first findings suggest that casein kinase 1ϵ, a closely related clock components implicated in period determination, could represent a novel target of pharmacological inhibition, thus stabilizing the circadian clock against phase shift (472). Finally, it is important to mention, that selective serotonine reuptake inhibitors (SSRI), as first-line treatment option for PTSD, have been shown to exert additional, CS-related effects. In particular, fluoxetine treatment was shown to modulate the CS *via* phase advances of SCN neuronal firing (514) and also normalize disrupted circadian locomotor activity and hippocampal clock gene expression in a genetic mouse model of high trait anxiety and depression (515).

## Conclusions

In Plato’s cosmology, as presented in the Timaeus, time is suggested to depend on the periodic regularity of movement, which is secured and defined by the planets (516). This periodic movement of our planet has contributed to the evolution of the internal time-keeping system, that creates and maintains cellular and systemic rhythmicity, through temporal organization of physiologic processes throughout several structural levels in the organism, the CS. The intrinsic rhythmicity of this system is based on a core set of clock genes involved with an autoregulatory transcriptional/translational feedback loop machinery. By rephrasing Plato’s words, we could, thus, state that human time depends on the periodic regularity of transcription, which is secured and defined by the clock genes. The award of the 2017 Nobel Prize in Physiology or Medicine to J.C. Hall, M. Rosbash and M. W. Young “for their discoveries of molecular mechanisms controlling the circadian rhythm” (517) is a testament to the fundamental importance of circadian clocks and the molecular complexity of behavior regulation.

However, over the past seven decades, modern society has cultivated a new, round-the-clock lifestyle, which enhances temporal misalignment between internal (i.e., central and peripheral) and geophysical circadian cycles. Given the close interconnection between the CS and the SS at various levels, internal desynchrony could synergistically contribute to the development of a higher stress sensitivity and vulnerability for stress-related disorders. Understanding the mechanisms susceptible to chronodisruption following toxic stress exposure and their role in a chronically dysregulated circadian network in stress-related disorders could provide new insights into disease mechanisms, advancing psychochronobiological treatment possibilities and enabling preventive strategies in stress-exposed populations (74, 312, 518).

## Author Contributions

AA managed all literature searches. AA and NN wrote the first draft of the paper. VB, GC, and PP contributed with significant text passages and revised the draft for important intellectual content. All authors have contributed to, read and approved the final version of the manuscript.

## Conflict of Interest

The authors declare that the research was conducted in the absence of any commercial or financial relationships that could be construed as a potential conflict of interest.
